# Protonation structure of the closed-cubane conformation of the O_2_-evolving complex in photosystem II

**DOI:** 10.1093/pnasnexus/pgac221

**Published:** 2022-10-03

**Authors:** Keisuke Saito, Hiroyuki Mino, Shunya Nishio, Hiroshi Ishikita

**Affiliations:** Department of Applied Chemistry, The University of Tokyo, 7-3-1 Hongo, Bunkyo-ku, Tokyo 113-8654, Japan; Research Center for Advanced Science and Technology, The University of Tokyo, 4-6-1 Komaba, Meguro-ku, Tokyo 153-8904, Japan; Division of Material Science, Graduate School of Science, Nagoya University, Furo-cho, Chikusa-ku, Nagoya 464-8602, Japan; Department of Applied Chemistry, The University of Tokyo, 7-3-1 Hongo, Bunkyo-ku, Tokyo 113-8654, Japan; Department of Applied Chemistry, The University of Tokyo, 7-3-1 Hongo, Bunkyo-ku, Tokyo 113-8654, Japan; Research Center for Advanced Science and Technology, The University of Tokyo, 4-6-1 Komaba, Meguro-ku, Tokyo 153-8904, Japan

## Abstract

In photosystem II (PSII), one-electron oxidation of the most stable state of the oxygen-evolving Mn_4_CaO_5_ cluster (S_1_) leads to the S_2_ state formation, Mn1(III)Mn2(IV)Mn3(IV)Mn4(IV) (open-cubane S_2_) or Mn1(IV)Mn2(IV)Mn3(IV)Mn4(III) (closed-cubane S_2_). In electron paramagnetic resonance (EPR) spectroscopy, the *g* = 4.1 signal is not observed in cyanobacterial PSII but in plant PSII, whereas the *g* = 4.8 signal is observed in cyanobacterial PSII and extrinsic-subunit-depleted plant PSII. Here, we investigated the closed-cubane S_2_ conformation, a candidate for a higher spin configuration that accounts for *g* > 4.1 EPR signal, considering all pairwise exchange couplings in the PSII protein environment (i.e. instead of considering only a single exchange coupling between the [Mn_3_(CaO_4_)] cubane region and the dangling Mn4 site). Only when a ligand water molecule that forms an H-bond with D1-Asp61 (W1) is deprotonated at dangling Mn4(IV), the *g* = 4.1 EPR spectra can be reproduced using the cyanobacterial PSII crystal structure. The closed-cubane S_2_ is less stable than the open-cubane S_2_ in cyanobacterial PSII, which may explain why the *g* = 4.1 EPR signal is absent in cyanobacterial PSII.

Significance Statement In natural photosynthesis, O_2_ evolves via the stepwise oxidation of the oxygen-evolving Mn_4_CaO_5_ center. The first-flash intermediate S_2_ state can be either the open-cubane Mn1(III)Mn2(IV)Mn3(IV)Mn4(IV) conformation, which is identified in the femtosecond X-ray free electron laser structure, or the closed-cubane Mn1(IV)Mn2(IV)Mn3(IV)Mn4(III) conformation. In electron paramagnetic resonance (EPR) spectroscopy, the low-spin *g* = 2 multiline and high-spin *g* > 4.1 signals were observed for the intermediate S_2_ state. The present quantum mechanical/molecular mechanical calculation reproduced the *g* = 4.1 EPR spectra only when a ligand water molecule that forms an H-bond with D1-Asp61 (W1) is deprotonated at dangling Mn4(IV). This will provide a basis for identification of higher oxidation-state conformations in the water oxidation mechanism.

Photosystem II (PSII) uses the Mn_4_CaO_5_ cluster as the catalytic center for water oxidation (1, 2). To increase the oxidation state S*_n_* (*n* = 0, 1, 2, and 3) of the Mn_4_CaO_5_ cluster and maximize the oxidation power, PSII uses the electron transfer pathway that proceeds via redox active D1-Tyr161 (TyrZ) to the oxidized chlorophyll pair, [P_D1_P_D2_]^•+^ [≈P_D1_^•+^ (3–6)]. Consequently, O_2_ evolves during the S_3_ to S_0_ transition. The crystal structures obtained using the femtosecond X-ray free electron laser (XFEL) suggest that a water molecule is incorporated into the O5 moiety of the Mn_4_CaO_5_ cluster during the S_2_ to S_3_ transition (7–9). The Mn_4_CaO_5_ cluster is composed of the three Mn sites in the cubane region (Mn1, Mn2, and Mn3) and the dangling Mn site (Mn4) (Fig. 1). The Mn valence state of S_1_ is Mn(III)_2_Mn(IV)_2_ in the high oxidation state model and Mn(III)_4_ in the low oxidation model (10). In the present study, if not otherwise specified, the S-states given refer to the high oxidation state model (11).

**Fig. 1.**
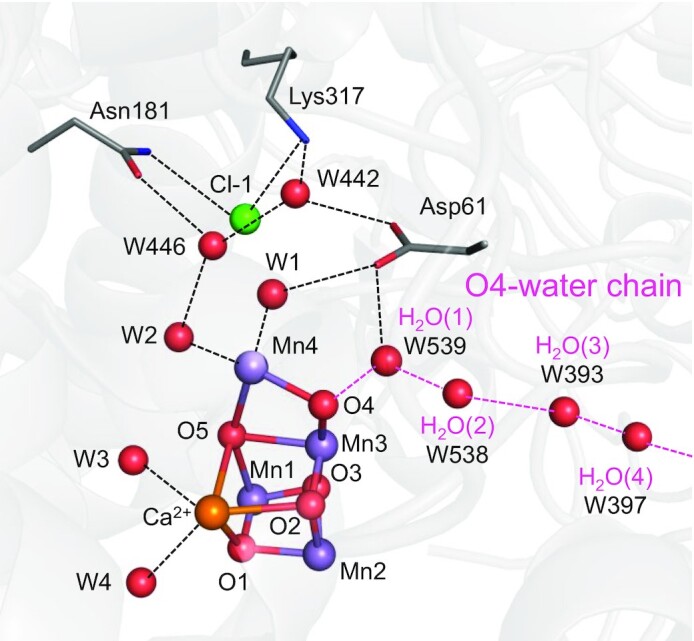
Overview of the Mn_4_CaO_5_ cluster and the main H-bond network. Dotted lines indicate H-bonds or electrostatic interactions. Pink dotted lines indicate the H-bond network of the O4-water chain.

As Mn2 and Mn3 are already oxidized to Mn(IV) in S_1_ according to the redox potential (6), either Mn1(III) or Mn4(III) is the oxidation site in the S_1_ to S_2_ transition. Mn1(IV). . .O5 is short and Mn4(III). . .O5 is long upon oxidation of Mn1(III) to Mn1(IV) (closed-cubane S_2_ conformation), whereas Mn1(III). . .O5 is long and Mn4(IV). . .O5 is short upon oxidation of Mn4(III) to Mn4(IV) (open-cubane S_2_ conformation) (12, 13). The open-cubane S_2_ conformation was identified in the XFEL structures, but not the closed-cubane S_2_ conformation (7–9), which may be due to the open-cubane S_2_ conformation being energetically more stable than the closed-cubane S_2_ conformation (14–17).

In electron paramagnetic resonance (EPR) spectroscopy for the Mn_4_CaO_5_ cluster, the *g* = 2 multiline and *g* > 4.1 signals are observed [e.g. (18)]. The *g* > 4.1 signals are classified into two cases: the *g* = 4.1 and *g* = 4.8 signals. The low-spin open-cubane and high-spin closed-cubane S_2_ conformations (12, 13) are likely to correspond to the *g* = 2 multiline and *g* = 4.1 signals, respectively. It should be noted that the *g* = 4.1 signal is distinct from the *g* = 4.8 signal. The *g* = 4.1 signal is observed in plant PSII, but not in cyanobacterial PSII under physiological conditions (19, 20), while the *g* = 4.8 signal is observed in cyanobacterial and extrinsic-subunit-depleted plant PSII. Taguchi et al. reported that the *g* = 4.1 signal was observed only in untreated spinach PSII, whereas the *g* = 4.7 to 4.9 signal was observed in cyanobacterial PSII [e.g. untreated and PsbO/V/U-depleted *Thermosynechococcus vulcanus* PSII and PsbO/V/U/Q’-depleted *Cyanidioschyzon merolae* PSII (21)], and PsbO/P/Q-depleted spinach PSII. This is consistent with the fact that in cyanobacterial PSII, the closed-cubane S_2_ is unstable with respect to the open-cubane S_2_ (14–17), and only the open-cubane S_2_ conformation was observed in the XFEL studies (7–9, 22).

The detailed character of the closed-cubane S_2_ conformation, which corresponds to the observed high-spin S_2_ (S = 5/2), is unclear. The EPR signals, including the *g* = 4.1 signal, are not fully reproduced in density functional theory (DFT) calculations (13, 23), which ignores the influence of the PSII protein environment (13, 23), as pointed out in ref. (24). Notably, the simulated EPR spectrum presented by Pantazis et al. (13) was obtained using a two-spin (Mn(IV)_3_, Mn4(III)) model [“*effective* coupling model” in ref. (13)], in which exchange couplings among the four Mn sites are simplified by a single exchange coupling between the Mn(IV)_3_ cubane region and the dangling Mn4(III) site, eventually ignoring all pairwise exchange couplings of the four-spin (Mn1, Mn2, Mn3, Mn4) model. However, it remains unexamined whether the simplified model *effectively* represents the relevant exchange couplings. Indeed, the calculated *g* value was 2.9 for the closed-cubane S_2_ conformation in the four spin (Mn1, Mn2, Mn3, Mn4) model as estimated from the simulated EPR spectrum shown in the Supplementary Material of ref. (13), which is not consistent with the experimentally measured *g* value of ∼4.1 (18). In DFT calculations by Pantazis et al., W2 was also assumed to be OH^–^ (13). Although W2 was repeatedly assumed to be OH^–^ in some studies (12, 13, 25–27), the reason is unclear. As far as we are aware, in 2011, Ames et al. already reported that W2 = OH^–^ is the most consistent model based on a comparison of the ground-state spin multiplicity, spin expectation values, and metal–metal distances (12). They also observed that H_2_O at W2 released the proton to OH^–^ at W3, leading to the formation of OH^–^ at W2. However, it remains unclear why they originally placed OH^–^ at W3 to expect the W2 deprotonation. In addition, the model was comprised of only the minimum components, Mn_4_CaO_5_ and the first sphere ligand groups. The model did not include any other protein components, e.g. the second sphere ligand residues (D1-Asp61 and CP43-Arg357) (12). Thus, the model cannot be considered to represent the Mn_4_CaO_5_ cluster in the PSII protein environment. In 2020, it was reported that p*K*_a_(W2) is the lowest in the closed-cubane S_2_ conformation in the absence of the PSII protein environment, whereas p*K*_a_(W1) is the lowest in the presence of the PSII protein environment, using a quantum chemical/molecular chemical (QM/MM) approach (28). The results also suggest that the low p*K*_a_(W2) value proposed by Ames et al. (12) is merely due to the absence of D1-Asp61, the H-bond acceptor of W1. W2 has no strong H-bond acceptor. Because W2 is H_2_O in the open-cubane S_2_ conformation with oxidized Mn4(IV) (28–31), it is more likely H_2_O in the closed-cubane S_2_ conformation with reduced Mn4(III).

In contrast, Fourier transform infrared (FTIR) spectra and theoretical calculations suggested that W2 was H_2_O in S_1_ and S_2_ (29). The PSII crystal structures (1), including the first flash-induced (1F) XFEL structure (corresponding to S_2_) (9), shows the presence of an H-bond between W1 and D1-Asp61, which supports the deprotonation of W1 rather than deprotonation of W2. Indeed, D1-Asp61 facilitates proton transfer (32–34), forming a low-barrier H-bond with W1 (28, 35). Notably, QM/MM-molecular dynamics studies by Narzi et al. suggested that proton-coupled electron transfer facilitated the OH^–^ formation at W1 in the closed-cubane S_2_ conformation in response to the release of the proton toward D1-Asp61 (32).

Alternatively, Corry and O’Malley proposed that the open-cubane S_2_ conformation with O4 = OH^–^ (and W2 = OH^–^) might correspond to a higher spin configuration on the basis of DFT calculations performed in the absence of the PSII protein environment (23). It remains unclear whether the proposal also holds true for PSII protein environment, since OH^–^ at O4 already releases the proton toward the O4-water chain during the S_0_ to S_1_ transition when W2 = H_2_O (36–38) ; H^–^ at O4 may be energetically unstable even in S_2_ with W2 = OH^–^. Neither the protonation structure nor the deprotonation site in the S_0_ to S_1_ transition was investigated in the presence of the PSII protein environment in ref. (23). Here, we investigated the origin of the *g* = 4.1 signal in EPR, using a QM/MM approach and considering the entire PSII protein environment.

## Methods

### Coordinates and atomic partial charges

The atomic coordinates were obtained from the X-ray diffraction (XRD) crystal structure of PSII monomer unit “A” of the PSII complexes from *Thermosynechococcus vulcanus* at a resolution of 1.9 Å (PDB code, 3ARC) (1). The closed-cubane S_2_ conformation was not observed in the 1F-XFEL structures (9, 22). In the present study, the initial coordinates of the closed-cubane S_2_ conformation was obtained, using the energetically more stable open-cubane S_2_ conformation (14–17) as an initial structure and analyzing the potential-energy profile for the O5 position between Mn1 and Mn4 in QM/MM calculations, as done previously (15). The resulting initial coordinates were again fully optimized in the absence of the O5 constraint in QM/MM calculations. Note that the structural difference in the Mn_4_CaO_5_ region between the XRD and 1F-XFEL is negligibly small, in particular, when the QM/MM optimized geometry is considered, since the calculated *E*_m_ values, which is sensitive to the difference in the electronic structure of the Mn_4_CaO_5_ cluster and the adjacent protein environment, are substantially the same (39). The heavy-atom positions were fixed while the H-atom positions were optimized with CHARMM (40). Water molecules in the crystal structures were represented explicitly as the flexible simple point-charge water model in the MM region. No other water molecules were added, as most of all water molecules adjacent to the Mn_4_CaO_5_ cluster are likely to have been identified due to the particularly low disorder in the region of the XRD structure (1). All titratable groups were ionized. D1-His337 was considered to be protonated (29). Atomic partial charges were obtained from the CHARMM22 (41) parameter set for amino acids and previous studies for cofactors (36), respectively.

### QM/MM calculations

The unrestricted DFT method was employed with the B3LYP functional and LACVP* basis sets [LANL2DZ (double ζ quality basis set with the Los Alamos effective core potential) for Mn and Ca atoms and 6–31G* for other atoms] (42) using the QSite (43) program. Counter ions were added to neutralize the system. In the QM region, all atomic coordinates were relaxed using the default geometry optimization algorism of QSite with the convergence criteria listed in Table S1. The results obtained using QSite were also evaluated by comparing with those obtained using ORCA (44) (e.g. Tables 2 and 3, see below). For the QM and MM regions, the van-der-Waals parameters of the OPLS2015 force field were used (45). In the MM region, the H-atom positions were energetically optimized, and the heavy-atom positions were fixed using the OPLS2005 force field, because the MM region is used mainly to reproduce (long-distance) electrostatic interactions with the QM region and the heavy-atom positions in the MM region should remain unchanged with respect to those in the original crystal structure. Thus, atoms on the QM surface at the QM/MM interface can be affected predominantly by H-bond or van-der-Waals-contact partners on the MM surface, without causing the unrealistic heavy-atom displacement of the MM region (artifact). The initial-guess wavefunctions were obtained using the ligand field theory (46) implemented in the QSite program. For the closed-cubane S_2_ conformation and the open-cubane S_2_ conformation in the protein environment, the QM region was defined as the Mn_4_CaO_5_ cluster (Mn_4_CaO_5_, the side-chains of D1-Asp170, D1-Glu189, D1-His332, D1-Glu333, D1-Asp342, and CP43-Glu354; the carboxy-terminal group of D1-Ala344; and water molecules, W1–W4), O4-water chain (W539, W538, and W393) (36, 37), Cl-1 binding site (Cl-1, W442, W446, and the side-chains of D1-Asn181 and D2-Lys317), second-sphere ligands (side-chains of D1-Asp61 and CP43-Arg357), and H-bond network of TyrZ (side-chains of D1-Tyr161, D1-His190, and D1-Asn298, W5, W6, and W7) (47, 48).

The QM/MM-optimized geometry was used as the initial geometry to analyze the potential energy profiles of the H-bond (e.g. O···H^+^··· O). The focusing H atom was moved along the O. . .O H-bond by 0.05 Å, after which the geometry was optimized by constraining the O–H^+^ and H^+^–O distances, and the energy was calculated.

### EPR spectrum calculations

The exchange coupling value, *J_ij_*, between Mn(*i*) and Mn(*j*) (*i, j* = 1, 2, 3, 4) were calculated using the broken symmetry approach (13, 49, 50). Under the classical spin approximation, the total energies of individual spin configurations in the closed-cubane S_2_ conformation, where (Mn1, Mn2, Mn3, Mn4) = (IV, IV, IV, III), are expressed in terms of the following equation (50):

(1) $$\begin{eqnarray*}
^{13/2}{E}_{\left( { \uparrow \uparrow \uparrow \uparrow } \right)} = - \left( {9/2} \right){J}_{12}\ - \left( {9/2} \right){J}_{13} - 6{J}_{14}\ - \left( {9/2} \right){J}_{23} - 6{J}_{24} - 6{J}_{34},
\end{eqnarray*}
$$

(2) $$\begin{eqnarray*}
^{7/2}{E}_{\left( { \uparrow \uparrow \downarrow \uparrow } \right)} = - \left( {9/2} \right){J}_{12} + {\rm{ }}\left( {9/2} \right){J}_{13} - 6{J}_{14} + \left( {9/2} \right){J}_{23} - 6{J}_{24} + 6{J}_{34},
\end{eqnarray*}
$$

(3) $$\begin{eqnarray*}
^{7/2}{E}_{\left( { \uparrow \downarrow \uparrow \uparrow } \right)} = \left( {9/2} \right){J}_{12} - \left( {9/2} \right){J}_{13} - 6{J}_{14} + \left( {9/2} \right){J}_{23} + 6{J}_{24} - 6{J}_{34},
\end{eqnarray*}
$$

(4) $$\begin{eqnarray*}
^{7/2}{E}_{\left( { \downarrow \uparrow \uparrow \uparrow } \right)} = \left( {9/2} \right){J}_{12} + \left( {9/2} \right){J}_{13} + 6{J}_{14} - \left( {9/2} \right){J}_{23} - 6{J}_{24} - 6{J}_{34},
\end{eqnarray*}
$$

(5) $$\begin{eqnarray*}
^{5/2}{E}_{\left( { \uparrow \uparrow \uparrow \downarrow } \right)} = - \left( {9/2} \right){J}_{12} - \left( {9/2} \right){J}_{13} + 6{J}_{14} - \left( {9/2} \right){J}_{23} + 6{J}_{24} + 6{J}_{34},
\end{eqnarray*}
$$

(6) $$\begin{eqnarray*}
^{1/2}{E}_{\left( { \downarrow \downarrow \uparrow \uparrow } \right)} = - \left( {9/2} \right){J}_{12} + \left( {9/2} \right){J}_{13} + 6{J}_{14} + \left( {9/2} \right){J}_{23} + 6{J}_{24} - 6{J}_{34},
\end{eqnarray*}
$$

(7) $$\begin{eqnarray*}
^{1/2}{E}_{\left( { \downarrow \uparrow \downarrow \uparrow } \right)} = \left( {9/2} \right){J}_{12} - \left( {9/2} \right){J}_{13} + 6{J}_{14} + \left( {9/2} \right){J}_{23} - 6{J}_{24} + 6{J}_{34},
\end{eqnarray*}
$$

(8) $$\begin{eqnarray*}
^{1/2}{E}_{\left( { \uparrow \downarrow \downarrow \uparrow } \right)} = \left( {9/2} \right){J}_{12} + \left( {9/2} \right){J}_{13} - 6{J}_{14} - \left( {9/2} \right){J}_{23} + 6{J}_{24} + 6{J}_{34},
\end{eqnarray*}
$$

where *^S^E*_sc_ is the total energy of the system for the total spin *S* and, sc is the spin configuration of (Mn1, Mn2, Mn3, Mn4) [e.g. (↑↓↓↑)], and *J_ij_* is the exchange interaction between the *i*-th and *j*-th ions. *^S^E*_sc_ was obtained from QM/MM calculations (Table S2). The pairwise *J* values were obtained as the best solution in the least-squares sense, solving the linear equations (eqs. 1 to 8) with the singular value decomposition (49). To calculate the total energy, the adiabatic approximation (i.e. using the optimized structures of all possible spin configurations) was performed (50). Note that the results were essentially the same when the vertical approximation (i.e. using the QM/MM-optimized geometry of the ground-state spin configurations) (50) was performed (Tables S3 and S4).

Spectral simulations were performed using the MATLAB R2019a software (The Mathworks, Inc) as done in previous studies (51). The effective Hamiltonian of the spin state of the Mn_4_CaO_5_ cluster can be expressed by

(9) $$\begin{eqnarray*}
\hat{\mathcal{H}} = \mathop \sum \nolimits_{i = 1}^4 \beta {{\boldsymbol{\hat{S}}}}_{\boldsymbol{i}} \cdot {{\boldsymbol{g}}}_{\boldsymbol{i}} \cdot {{\boldsymbol{B}}}_{\boldsymbol 0} + \sum {{\boldsymbol{\hat{I}}}}_{\boldsymbol{i}} \cdot {{\boldsymbol{A}}}_{\boldsymbol{i}} \cdot {{\boldsymbol{\hat{S}}}}_{\boldsymbol{i}} + {\hat{\mathcal{H}}}_{{\rm{ZFS}}} + {\hat{\mathcal{H}}}_{{\rm{ex}}},
\end{eqnarray*}
$$

where ${{\boldsymbol{\hat{S}}}}_{\boldsymbol{i}}$, and ${{\boldsymbol{\hat{I}}}}_{\boldsymbol{i}}$ are the operators of electron spin and nuclear spin of the *i*-th Mn ion, respectively, ***g_i_*** is the *g*-tensor of the *i*-th Mn ion, ***A_i_*** is the effective hyperfine tensor of the *i*-th Mn ion, and *β* is the Bohr magneton. Here, ***g_i_*** is approximated to be isotropic and independent of Mn*i* (*g_i_* = 2). ${\hat{\mathcal{H}}}_{{\rm{ZFS}}}$ and ${\hat{\mathcal{H}}}_{{\rm{ex}}}$ are Hamiltonians of the zero-field splitting (ZFS) and the exchange, respectively, and are espressed as

(10) $$\begin{eqnarray*}
{\hat{\mathcal{H}}}_{{\rm{ZFS}}} = {{\boldsymbol{\hat{S}}}}_{{{\bf total}}} \cdot {\boldsymbol{D}} \cdot {{\boldsymbol{\hat{S}}}}_{{{\bf total}}},
\end{eqnarray*}
$$

(11) $$\begin{eqnarray*}
{\hat{\mathcal{H}}}_{{\rm{ex}}} = - \mathop \sum \nolimits_{i < j} 2{J}_{ij}{{\boldsymbol{\hat{S}}}}_{\boldsymbol{i}} \cdot {{\boldsymbol{\hat{S}}}}_{\boldsymbol{j}},
\end{eqnarray*}
$$

with

(12) $$\begin{eqnarray*}
{{\boldsymbol{\hat{S}}}}_{{{\bf total}}} = \mathop \sum \nolimits_{i = 1}^4 {{\boldsymbol{\hat{S}}}}_{\boldsymbol{i}},
\end{eqnarray*}
$$

where ***D*** is the ZFS tensor of the cluster, *J_ij_* is the exchange interaction between the *i*-th and *j*-th Mn ions. Using the principle axes *x, y*, and *z* of ***D***, ${\hat{\mathcal{H}}}_{{\rm{ZFS}}}$ is expressed as

(13) $$\begin{eqnarray*}
{\hat{\mathcal{H}}}_{{\rm{ZFS}}} = D\left[ {\hat{S}_{{\rm{total}},z}^2 - \frac{1}{3}{{\hat{S}}}_{{\rm{total}}}^2} \right] + E\left( {\hat{S}_{{\rm{total}},x}^2 - \hat{S}_{{\rm{total}},y}^2} \right),
\end{eqnarray*}
$$

where *D* and *E* are ZFS parameters, which are obtained from the principle value of ***D*_total_**, and $\hat{S}_{{\rm{total}},p}^2$ is the operator of the *p* (*x, y*, or *z*) component of ${{\boldsymbol{\hat{S}}}}_{{{\bf total}}}$.

The model Hamiltonian (eqs. 9, 11, and 13) is used in the simulations. Here, Mn1, Mn2, and Mn3 are Mn(IV) (*S*_1_ *= S*_2_ *= S*_3_ = 3/2), and Mn4 is Mn(III) (*S*_4_ = 2) with *D* = –0.445 and *E*/*D* = 0.25 (52). Diagonalizing $\hat{\mathcal{H}}$, we obtain the eigen energy *E_n_*(***B*_0_**) of the *n*-th state |*n*(***B*_0_**)> as a function of ***B*_0_**, where the hyperfine splitting term is not included (51). The transition probability *P_k_^n^* from the initial state |*n*(***B*_0_**)> to the final state |*k*(***B*_0_**)> is given by the following equation based on the Fermi’s golden rule,

(14) $$\begin{eqnarray*}
P_k^n = \frac{2\pi}{\hbar}\delta (\left|E_k({\boldsymbol B_0}) - E_n({\boldsymbol B_0})\right| - {\hbar \omega})\left | \lt k({\boldsymbol B_0}) \right |\beta g {\hat{\boldsymbol S}_{\boldsymbol total}} \cdot {\boldsymbol B_1} \left | n({\boldsymbol B_0}) \gt \right|^2,\nonumber\\
\end{eqnarray*}
$$

where ${{\boldsymbol{\hat{S}}}}_{{{\bf total}}}$ is the operator of the total spin of the system and ***B*_1_** is the magnetic field of the external microwave with the angular frequency ω and ω is set to be 9.50 GHz. Assuming that the hyperfine interactions are approximated by using an isotropic Gaussian, the δ function in eq. 14 is replaced with the spectral lineshape of

(15) $$\begin{eqnarray*}
\rho _k^n\left( {\omega ,{{\boldsymbol{B}}}_{\boldsymbol 0}} \right) = \frac{1}{{\sqrt {2\pi {{\rm{\Delta }}}^2} }}\exp \left[ { - \frac{{{{\{ \left| {{E}_k\left( {{{\boldsymbol{B}}}_{\boldsymbol 0}} \right) - {E}_n\left( {{{\boldsymbol{B}}}_{\boldsymbol 0}} \right)} \right| - \hbar \omega \} }}^2}}{{2{{\rm{\Delta }}}^2}}} \right],
\end{eqnarray*}
$$

where *Δ* is the width and is set to be 0.033 cm^−1^ (corresponding to 350 G). Then, the following equation was obtained:

(16) $$\begin{eqnarray*}
P_k^n({{\boldsymbol B}_{\boldsymbol 0}},{{\boldsymbol B}_{\boldsymbol 1}}) = \frac{2\pi}{\hbar }\rho_k^n({\omega} ,{{\boldsymbol B}_{\boldsymbol 0}}) \times \left|\lt {k({{\boldsymbol B}_{\boldsymbol 0}})} \right|\beta g{\hat{\boldsymbol S}}_{\boldsymbol total} \cdot {{\boldsymbol B}_{\boldsymbol 1}}\left| {n({{\boldsymbol B}_{\boldsymbol 0}})} \gt \right|^2.
\end{eqnarray*}
$$

Taking the integral over all directions of ${{\boldsymbol{B}}}_{\boldsymbol 0}$ and ${{\boldsymbol{B}}}_{\boldsymbol 1}$ (${{\boldsymbol{B}}}_{\boldsymbol 0} \ \bot \ {{\boldsymbol{B}}}_{\boldsymbol 1}$), the absorption spectrum *I*(*B*_0_) as a function of *B*_0_ can be calculated by

(17) $$\begin{eqnarray*}
I\left( {{B}_{0{\rm{\ }}}} \right) = C\smallint {\rm{sin}}\theta d\theta d\phi \smallint d\alpha \mathop \sum \nolimits_{k,n} P_k^n\left( {R\left( {\theta ,\ \phi } \right){{\boldsymbol{B}}}_{{\boldsymbol 0}{\boldsymbol{z}}},R\left( {\theta ,\ \phi } \right){{\boldsymbol{B}}}_{{\boldsymbol{1xy}}}\left( \alpha \right)} \right),\nonumber\\
\end{eqnarray*}
$$

with ${{\boldsymbol{B}}}_{{\boldsymbol 0}{\boldsymbol{z}}} = {\ }^t[ {0,0,{B}_0} ]$, ${{\boldsymbol{B}}}_{{\boldsymbol{1xy}}}( \alpha ) = {\ }^t[ {{B}_1{\rm{cos}}\alpha ,{B}_1{\rm{sin}}\alpha ,0} ]$,

$$\begin{equation*}
R\left( {\theta ,\ \phi } \right) = \left[ {\begin{array}{@{}*{3}{c}@{}} {{\rm{cos}}\phi }&{ - {\rm{sin}}\phi }&0\\ {{\rm{cos}}\theta {\rm{sin}}\phi }&{{\rm{cos}}\theta {\rm{sin}}\phi }&{ - {\rm{sin}}\theta }\\ {{\rm{sin}}\theta {\rm{sin}}\phi }&{{\rm{sin}}\theta {\rm{cos}}\phi }&{{\rm{cos}}\theta } \end{array}} \right],
\end{equation*}
$$

where *α* is an angle and *θ* and *ϕ* are the angles defined in the polar coordinates and the summation takes over all states considered.

## Results and discussion

The following five closed-cubane S_2_ conformations were obtained in QM/MM calculations: (1) two cases for W1 = OH^–^ and W2 = H_2_O: (1a) the axis of the *dz*^2^ orbital on the Mn4 site is either [D1-Asp170. . .Mn4. . .D1-Glu333] or (1b) [O4. . .Mn4. . .W2] (Fig. S1a, b); (2) two cases for W1 = H_2_O and W2 = OH^–^: (2a) W2 donates an H-bond to either W446 or (2b) O5 (Fig. S1c, d); (3) one case for W1 = H_2_O and W2 = H_2_O (summarized in Table 1).

**Table 1.** Calculated values for exchanging coupling *J* (cm^–1^) and *g*. –: not applicable (or not determined).

**Ligand** **Mn4-*dz^2^* axis^a^**	***J*_12_**	***J*_13_**	***J*_14_**	***J*_23_**	***J*_24_**	***J*_34_**	***J*_14+24+34_**	***J*_eff_^b^**	**ZFS^c^**		***g***	***S*** ^**d**^
**[this study] four-spin model**												
**W1: OH^–^, W2: H_2_O** (D170-Mn-E333 axis)	24.1	7.6	1.0	24.2	1.8	−44.6	−41.8	–	totalMn4 site	→→	**4.0** **3.7**	5/2
												
**W1: OH^–^, W2: H_2_O**(O4-Mn-W2 axis)	23.7	8.6	−0.9	22.3	4.9	−28.9	−24.9	–	totalMn4 site	→→	**4.0** **3.6**	5/2
												
**W1: H_2_O, W2: OH^–^ (W2...O_W446_)**(O4-Mn-W2 axis)	29.9	8.4	8.8	24.0	4.8	−18.1	−4.5	–			–	5/2
												
**W1: H_2_O, W2: OH^–^ (W2...O_W5_)**(O4-Mn-W2 axis)	26.4	5.7	6.9	24.3	−0.4	−9.9	−3.4	–			–	5/2
												
**W1: H_2_O, W2: H_2_O**(O5-Mn-W1 axis)	26.7	−0.2	6.3	31.2	0.8	−1.2	5.9	–			–	11/2
**[Pantazis et al.] four-spin model** ^e^												
**W1: H_2_O, W2: OH^–^**	30.5	13.0	0^g^	35.5	0^g^	−7.6	−7.6	–	Mn4 site	→	**2.9** ^f^	5/2
**[Pantazis et al.] effective two-spin model** ^e^												
**(no geometry)**	–	–	–	–	–	–	–	−**2.3**	Mn4 site	←	2.9^f^	
**(no geometry)** ^i^	–	–	–	–	–	–	–	(−**10.6**	Mn4 site	←	∼4)^h^	

Arrows indicate the relationship between *J* and *g*. That is, → indicates that *J* is input and *g* is output, whereas ← indicates that *g* is input and *J* is output. The resulting output values are in bold.

a See Fig. S1.

b A parameter to approximate the four-spin model as the two-spin model in ref. (13).

c ZFS scheme.

d Spin *S* in the ground state.

e Ref. (13).

f See Fig. S2.

g See Table S3 in ref. (13).

h *g* ∼ 4 was not obtained in ref. (13), as *J*_eff_ obtained from the calculated *J* values was –2.3 cm^–1^ [Fig. S5 in ref. (13)]. To reproduce *g* ∼ 4 in the two-spin model, |*J*_eff_| must be > 10.6.

i *J*_eff_ = –10.6 cm^–1^ was obtained from the experimentally measured value of *g* ∼ 4. Note that *g* ∼ 4 was not obtained in ref. (13).

### EPR spectra for the W1 and W2 protonation states

**Fig. 2.**
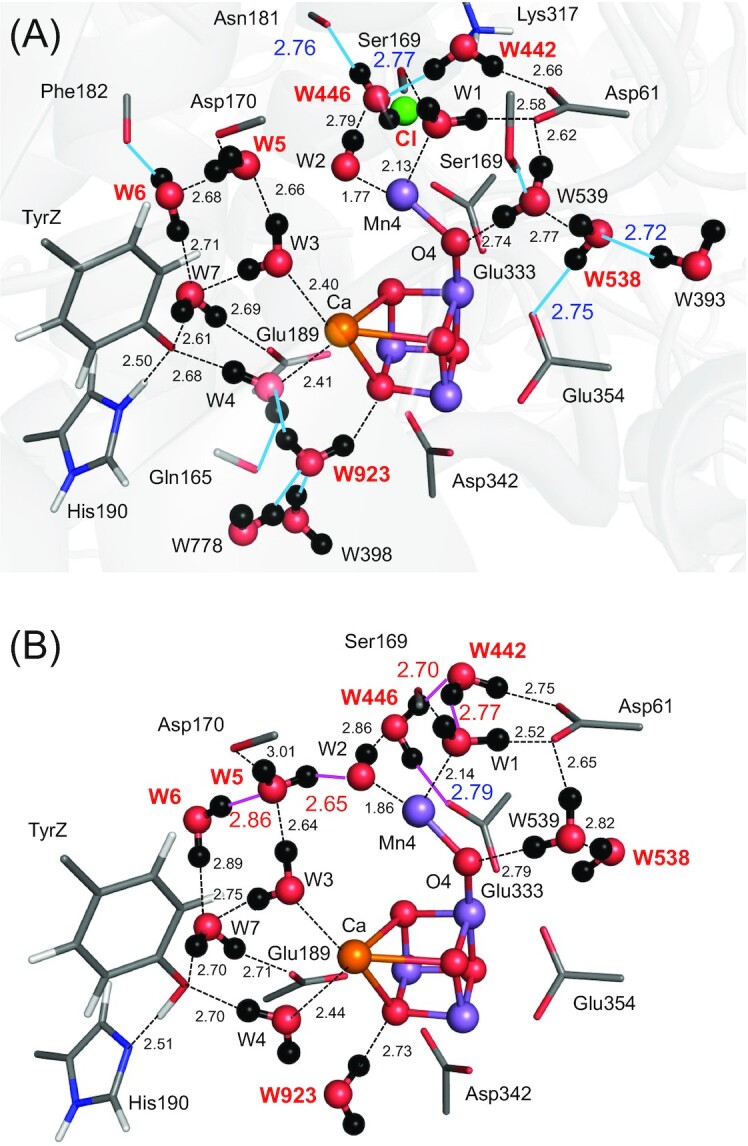
H-bond network of the closed-cubane S_2_ conformation with W2 = OH^–^. (A) This study (QM/MM calculations). The protein environment is gray-colored. (B) DFT calculations by Pantazis et al. (13) (i.e. in the absence of the PSII protein environment). Groups that show the significant difference between the two calculations are red labeled. Cyan lines indicate H-bonds that disappear in the DFT calculation mainly owing to the absence of the PSII protein environment. Pink lines indicate H-bonds that form only in the DFT calculation.

The values of exchange coupling *J* were calculated based on these five QM/MM-optimized geometries (Fig. 2A). The calculated *J* values depend on the protonation states of W1 and W2 (Table 1). In particular, the magnitude of exchange *J* coupling between Mn3 and Mn4 (|*J*_34_|) is significantly large (>∼30 cm^–1^) when W1 = OH^–^ (discussed later).

**Fig. 3.**
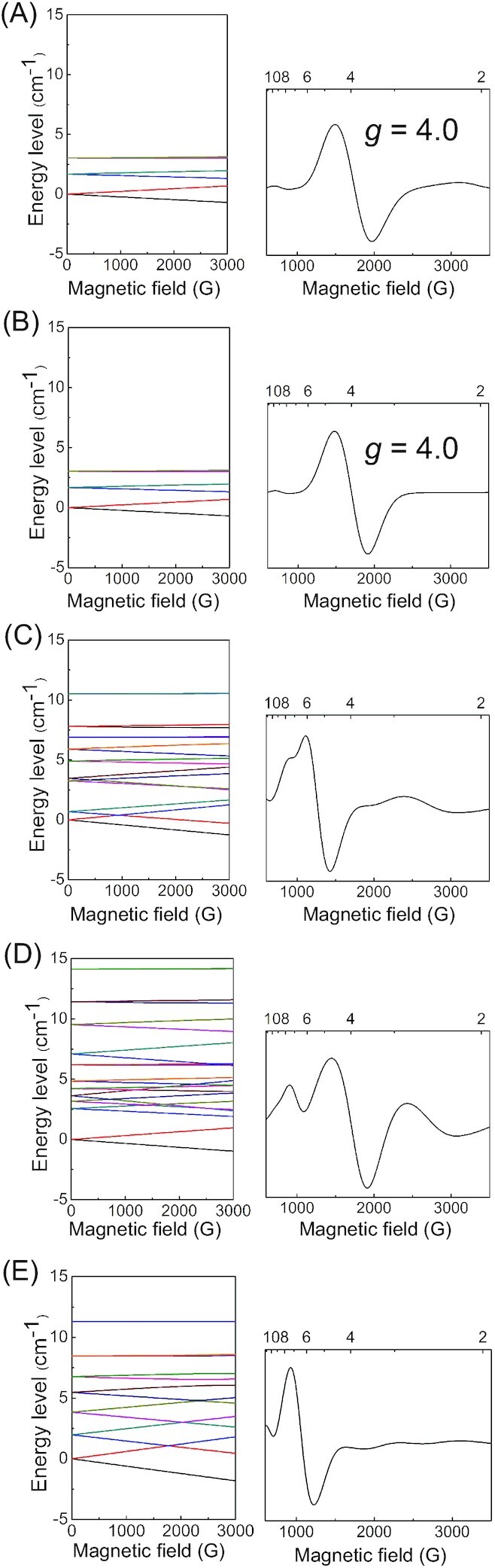
Simulated EPR spectra and energy level *E*_n_(***B*_0_**) when ***B*_0_** is parallel to the principle *z* axis of the ***D*** (*B*_0_//*D*) with *D* = –0.445 cm^–1^. (A) W1 = OH^–^ and W2 = H_2_O with the Mn4-*dz*^2^ axis [D170. . .Mn4. . .E333]. (B) W1 = OH^–^ and W2 = H_2_O with the Mn4-*dz*^2^ axis [O4. . .Mn4. . .W2]. (C) W1 = H_2_O and W2 = OH^–^. . .O_W446_ with the Mn4-*dz*^2^ axis [O4. . .Mn4. . .W2]. (D) W1 = H_2_O and W2 = OH^–^. . .O_W5_ with the Mn4-*dz*^2^ axis [O4. . .Mn4. . .W2]. (E) W1 = H_2_O and W2 = H_2_O with the Mn4-*dz*^2^ axis [O5. . .Mn4. . .W1]. The EPR transitions (A to E) are calculated by considering the lowest 6, 6, 18, 18, and 14 sublevels, respectively.

**Fig. 4.**
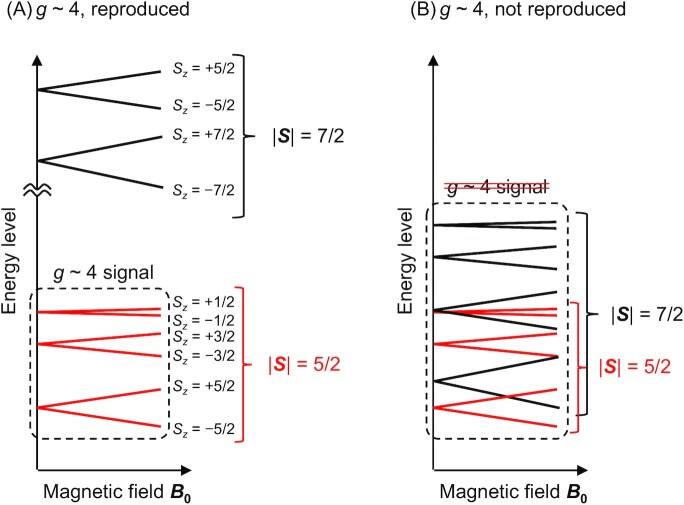
Typical energy levels for the spin states with |***S***| = 5/2 (i.e. including *S_z_* = ±1/2, ±3/2, and ± 5/2) and the spin states with |***S***| = 7/2 (i.e. including *S_z_* = ±1/2, ±3/2, ±5/2, and ± 7/2). (A) Energy level that can reproduce the *g* ∼ 4 signal. The |***S***| = 5/2 system, which is comprised of *S_z_* = ±1/2, ±3/2, and ± 5/2 states, is isolated from the |***S***| = 7/2 system, which is comprised of *S_z_* = ±1/2, ±3/2, ±5/2, and ± 7/2 states. The first three energy levels at the magnetic field ***B***_0_ = 0 correspond to the spin states with |***S***| = 5/2, whereas the fourth-lowest energy level corresponds to the spin states with |***S***| = 7/2. The *g* ∼ 4 signal can be reproduced because the *g* ∼ 4 signal originates from the isolated |***S***| = 5/2 system (13). (B) Energy level that cannot reproduce the *g* ∼ 4 signal. The |***S****|* = 5/2 system is not isolated from the |***S***| = 7/2 system because the difference in energy is not sufficiently larger than the splitting range.

Using all six pairwise *J* values, the energy levels that correspond to the EPR spectrum were calculated (Fig. 3). The *g* ∼ 4 EPR spectrum originates from the isolated |***S****|* = 5/2 system, which is comprised of *S_z_* = ±1/2, ±3/2, and ± 5/2 states (13) (Fig. 4A). When W2 = OH^–^ (Figs. 3C and D), which was assumed in ref. (13), the energy level of the first excited spin state (*S* = 13/2; i.e. ^13/2^*E* in eq. 1) is too low (27 to 40 cm^–1^; Table S2) in comparison with that of the ground spin state (*S* = 5/2; i.e. ^5/2^*E* in eq. 5) to reproduce the *g* ∼ 4 EPR spectrum (e.g. see the fourth-lowest energy at the magnetic field ***B***_0_ = 0 in Figs. 3 and 4B). For the same reason, the EPR spectrum was also not reproduced when W1 = H_2_O and W2 = H_2_O (Fig. 3E).

In contrast, the *g* ∼ 4 EPR spectrum is reproduced only when W1 = OH^–^ and W2 = H_2_O (Fig. 3A and B), which is consistent with QM/MM-molecular dynamics studies by Narzi et al. that showed the OH^–^ formation at W1 in the closed-cubane S_2_ conformation in response to the release of the proton toward D1-Asp61 (32). This is because the energy level of the first excited spin state (*S* = 7/2; i.e. ^7/2^*E* in eqs. 2 to 4) is sufficiently higher (229 to 253 cm^–1^; Table S2) than that of the ground spin state (*S* = 5/2; i.e. ^5/2^*E* in eq. 5) and the energy levels in the |***S***| = 5/2 system (Fig. 4A) are sufficiently separated from those in the |***S****|* = 7/2 system (Fig. 4A). The calculated *g* values of 4.0 reproduce the experimentally measured *g* value of 4.1 for the high-spin S_2_ state more appropriately than the previously calculated *g* values of 2.9 (Fig. S2) for W1 = H_2_O and W2 = OH^–^ in ref. (13) (Table 1). Note that calculated *J* values do not vary significantly with basis set and functional (Table 2).

**Table 2.** Calculated values for exchanging coupling *J* (cm^–1^) for the closed-cubane S_2_ conformation with W2 = OH^–^ in the absence of the PSII protein environment, using the atomic coordinates in ref. (13). –: not applicable (or not determined).

***J* values**	***J*_12_**	***J*_13_**	***J*_14_**	***J*_23_**	***J*_24_**	***J*_34_**	**Functional**	**Basis set**	**Package**
Pantazis et al.	30.5^a^	13.0^a^	–^a^	35.5^a^	–^a^	−7.6^a^	BP86	TZVP (Mn, O, N) SARC-SVP (others)	ORCA
This study	27.8	13.0	4.6	31.8	2.0	−6.5	B3LYP	LANL2DZ (Mn, Ca) 6–31G* (others)	Jaguar

a See ref. (13).

In cyanobacterial PSII, the Mn_4_CaO_5_ cluster exists as the open-cubane S_2_ conformation [e.g. as observed in the XFEL structures (7–9)], because the closed-cubane structure is significantly unstable with respect to the open-cubene structure (14–17). This is consistent with the fact that (i) the *g* = 4.1 EPR spectrum (not the *g* = 4.8 spectrum) was reproduced for the closed-cubene S_2_ conformation (Fig. 3) and (ii) the *g* = 4.1 signal is not observed in cyanobacterial PSII (19–21).

### ZFS scheme

**Fig. 5.**
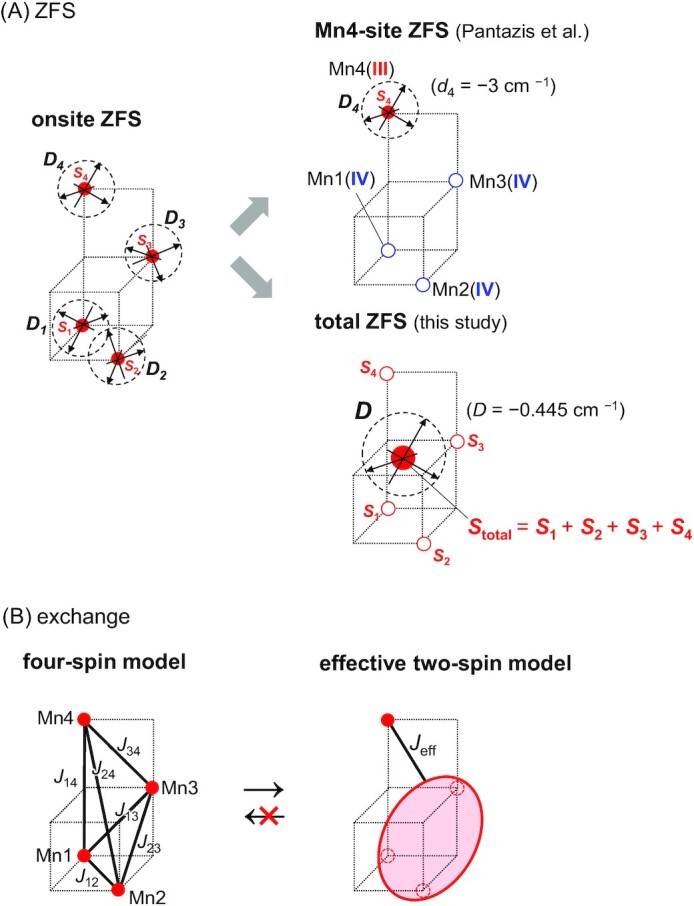
Schematic models of spin Hamiltonian. (a) ZFS Hamiltonian (${\hat{\mathcal{H}}}_{{\rm{ZFS}}}$). The onsite-ZFS scheme (described by four individual anisotropic spin systems [*S*_1_, *S*_2_, *S*_3_, and *S*_4_]; eq. 18) is approximated by the Mn4-site-ZFS scheme (predominantly expressed by the Mn4 spin *S*_4_; eq. 19) or the total-ZFS scheme (expressed by the single total spin *S*_total_; eq. 10). The Mn4-site-ZFS scheme was used in ref. (13) (b) Exchange Hamiltonian (${\hat{\mathcal{H}}}_{{\rm{ex}}}$). The four-spin model (considering all pairwise *J* values; eq. 11) is approximated by the effective two-spin model (considering only an exchange coupling *J*_eff_ between the [Mn1, Mn2, Mn3] cubane region and the dangling Mn4 site).

In the present EPR spectrum simulation, the ZFS Hamiltonian ${\hat{\mathcal{H}}}_{{\rm{ZFS}}}$ is described as (eq. 10) based on “the **total-ZFS** scheme”, where the Mn_4_CaO_5_ cluster is regarded as a single anisotropic spin system ${{\boldsymbol{\hat{S}}}}_{{{\bf total}}}$ (Fig. 5A). On the other hand, in “the **onsite-ZFS** scheme”, ${\hat{\mathcal{H}}}_{{\rm{ZFS}}}$ is expressed as

(18) $$\begin{eqnarray*}
{\hat{\mathcal{H}}}_{{\rm{ZFS}}} = \mathop \sum \nolimits_{i \geq j}^4 {{\boldsymbol{\hat{S}}}}_{\boldsymbol{i}} \cdot {{\boldsymbol{D}}}_{{\boldsymbol{ij}}} \cdot {{\boldsymbol{\hat{S}}}}_{\boldsymbol{j}},
\end{eqnarray*}
$$

with the onsite-ZFS tensor ***D_ij_*** between the Mn*i* and Mn*j* sites. Assuming that the ZFS effect induced by the spin dipoles is negligi-ble of two distinct Mn sites (i.e. ${{\boldsymbol{D}}}_{{\boldsymbol{ij}}}$ = 0 for *i* ≠ *j*), ${\hat{\mathcal{H}}}_{{\rm{ZFS}}}$ can be recast as

(19) $$\begin{eqnarray*}
{\hat{\mathcal{H}}}_{{\rm{ZFS}}} = \mathop \sum \nolimits_{i\ = {\rm{\ }}1}^4 {{\boldsymbol{\hat{S}}}}_{\boldsymbol{i}} \cdot {{\boldsymbol{D}}}_{\boldsymbol{i}} \cdot {{\boldsymbol{\hat{S}}}}_{\boldsymbol{i}},
\end{eqnarray*}
$$

with the onsite-ZFS tensor ***D_i_*** (= ***D_ii_***) for each Mn*i* site. Here, the Mn_4_CaO_5_ cluster is regarded as a complex of four individual anisotropic spin systems [Mn1, Mn2, Mn3, and Mn4] (Fig. 5A, left panel). Pantazis et al. simulated the EPR spectra based on the onsite-ZFS scheme using the following approximation (**Mn4-site-ZFS scheme**; Fig. 5A): they assumed that the ZFS tensor is determined predominantly by the Mn4 spin [***D*_1_, *D*_2_**, and ***D*_3_** for Mn1(IV), Mn2(IV), and Mn3(IV) are zero, but ***D*_4_** for Mn4(III) has a nonzero value], i.e.

(20) $$\begin{eqnarray*}
{\hat{\mathcal{H}}}_{{\rm{ZFS}}} \cong {{\boldsymbol{\hat{S}}}}_{\boldsymbol 4} \cdot {{\boldsymbol{D}}}_{\boldsymbol 4} \cdot {{\boldsymbol{\hat{S}}}}_{\boldsymbol 4} = {d}_4\left[ {\hat{S}_{4,z}^2 - \frac{1}{3}\hat{S}_4^2} \right] + {e}_4\left( {\hat{S}_{4,x}^2 - \hat{S}_{4,y}^2} \right),
\end{eqnarray*}
$$

where *d*_4_ and *e*_4_ are onsite ZFS parameters derived from the principle value of ***D*_4_**, and $\hat{S}_{4,p}^2$ is the operator of the *p* (*x, y*, or *z*) component of ${{\boldsymbol{\hat{S}}}}_4$.

This approximation may be rationalized when the onsite-ZFS tensor ***D_i_*** for Mn(IV) is negligibly smaller than that for Mn(III) (53). Note that to the best of our knowledge, large ***D_i_*** is not reported for Mn(IV) in the Mn(III)Mn(IV) complexes, while ***D_i_*** for Mn(IV) is not necessarily small in mononuclear Mn complexes (54) (see below).

In the present study, the EPR spectra were also calculated using the original Mn4-site-ZFS parameter as used in ref. (13). The results obtained in the present study are similar to those in the total-ZFS scheme: only when W1 = OH^–^ and W2 = H_2_O, the calculated *g* values are 3.6 to 3.7 (Table 1 and Fig. S3) and reproduce the experimentally measured *g* value of 4.1 more appropriately than the previously calculated *g* values of 2.9 in ref. (13) (Table 1).

Thus, in contrast to the previous study (13) (*g* = 2.9; Fig. S2) for W1 = H_2_O and W2 = OH^–^, the present EPR spectrum simulation with W1 = OH^–^ and W2 = H_2_O reproduces the *g* ∼ 4 signal appropriately (Figs. 3A, B and S1a, b, and Table 1) in both the total- and Mn4-site-ZFS schemes.

### Comparison between the total- and Mn4-site-ZFS schemes

It is unclear whether the Mn4-site-ZFS scheme is justified, as (i) the ZFS effect (***D_ij_***) induced by spin dipoles of two distinct Mn sites may not be negligible and (ii) the onsite-ZFS tensor ***D_i_*** is not necessarily negligibly small even for Mn(IV) (54). In addition, (iii) EPR studies suggested that the *z* axis of the ZFS tensor (i.e. experimentally measured *z* axis) was oriented along the Mn4-O5 direction independently of the Mn4 valence (51). In the Mn4-site-ZFS scheme, the experimentally measured *z* axis should correspond to the *z* axis of the Mn4-site-ZFS tensor ***D_4_*** (*D*_4_-*z* axis). However, the direction of the experimentally measured *z* axis (51) may not be consistent with the direction of the *D*_4_*-z* axis, because the *D*_4_-*z* axis is directly associated with the Mn4-*dz^2^* axis (53), which is not always oriented along the Mn4-O5 direction (Table 1).

In contrast, in the total-ZFS scheme, the orientation of the *z* axis of the total-ZFS tensor***D*** (*D*-*z* axis) originates from not only the Mn4-*dz*^2^ axis but also the entire electronic structure of the Mn_4_CaO_5_ cluster and can be oriented along the different direction from the Mn4-*dz^2^* axis. Accordingly, the *D*-*z* axis is in line with the experimentally measured *z* axis (51). In the onsite-ZFS scheme (eq. 20), the *D*_4_*-z* axis is unlikely to represent the *D*-*z* axis in the total-ZFS scheme, probably because (i) the *d*_4_ value is not sufficiently accurate and (ii) the ZFS effect (***D_ij_***) induced by spin dipoles of two distinct Mn sites is not negligible.

Overall, the conclusion that the present EPR spectrum simulation with W1 = OH^–^ and W2 = H_2_O reproduces the *g* ∼ 4 signal (Fig. 3A, B and Fig. S3a, b) appropriately is valid regardless of the total- and Mn4-site-ZFS schemes. Because the experimentally measured *z* axis is not consistent with the EPR spectrum simulation based on the Mn4-site-ZFS scheme, the total-ZFS scheme seems to be more appropriate than the Mn4-site-ZFS scheme.

### Comparison with previous studies: closed-cubane S_2_ conformation with W2 = OH^–^

In previous studies by Pantazis et al. (13), the simulated EPR spectrum was eventually obtained using effective two-spin models. In ref. (13), the actual *g* value was calculated using six pairwise *J* values in the “four”-spin model is 2.9, not >4 (Table 1 and Fig. S2). In ref. (13), to approximate the four-spin model as the simplified two-spin model, a parameter “*J*_eff_” was introduced (Fig. 5B). *g* = 2.9, calculated using pairwise *J* values in the four-spin model (i.e. DFT calculation), corresponds to *J*_eff_ = –2.3 cm^–1^ in the simplified two-spin model [Table 1 and Fig. S5 in ref. (13)]. If *J*_eff_ = –10.6 cm^–1^ were obtained, *g* ∼ 4 might have been reproduced (Table 1). However, this is not the case with their DFT calculations (i.e. *J*_eff_ = –2.3 cm^–1^) (13). Since it is impossible to reconstruct a four-spin model from the two-spin model (Fig. 5B), there is no corresponding Mn_4_CaO_5_ cluster geometry with *J*_eff_ = –10.6 cm^–1^. (Note: their simulated EPR spectrum shown in Fig. 4 in ref. (13) was a typical spectrum obtained merely from one arbitrary atom with *S* = 5/2, not from the original/quantum-chemically optimized Mn_4_CaO_5_ geometry in the crystal structure.) Thus, no EPR spectrum with *g* ∼ 4, which was calculated using “*all six pairwise J values* (13)” with W2 = OH^–^ in the relevant PSII structure (see below), was provided in ref. (13).

In ref. (13), the geometry was obtained in the absence of the PSII protein environment: the protein backbone and Cl-1 were also absent. The absence of the PSII protein environment leads to irrelevant structural changes as an artifact. In ref. (13), W446 at Cl-1 donates an H-bond to one of the carboxyl O sites of D1-Glu333 due to the absence of anionic Cl-1, which weakens the ligand interaction between D1-Glu333 and Mn4. Thus, the stabilization of oxidized Mn4(IV) by D1-Glu333 is underestimated (i.e. the stability of the open-cubane S_2_ conformation is underestimated). The absence of Cl-1, which is closer to Mn4 (6.7 Å) than Mn1 (7.9 Å), also underestimates the stability of Mn(IV) (17). This explains why the closed-cubane and open-cubane S_2_ conformations are isoenergetic in ref. (13) in contrast to other studies (14–17). The absence of the protein backbone in ref. (13) also induces alteration of the H-bond pattern in a cluster of water molecules near TyrZ (i.e. W3, W5, W6, and W7, Fig. 2) as an artifact. The absence of the protein backbone of D1-Phe182 makes W6 donate an H-bond to W5, which induces the H-bond donation of W5 to W2, stabilizing OH^–^ at W2 as an artifact (Fig. 2B). Thus, the conclusion of W2 = OH^–^ in the closed-cubane conformation (13) needs to be revisited.

### Comparison with previous studies: open-cubane S_2_ conformation with O4 = OH^–^

**Fig. 6.**
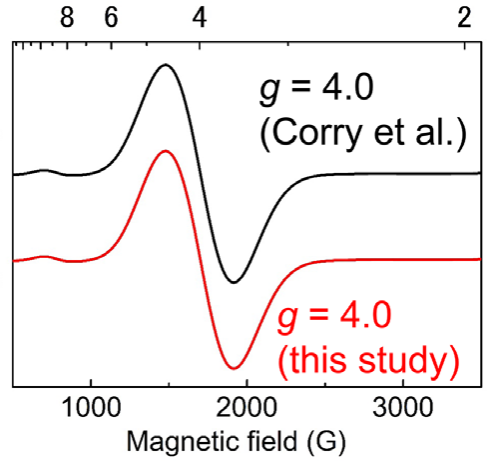
Simulated EPR spectra for the open-cubane S_2_ conformation with O4 = OH^–^ in the absence of the PSII protein environment, using the atomic coordinates in ref. (23) (i.e. W2 = OH^–^). Black and red curves indicate EPR spectra simulated using the *J* values of Corry et al. and this study listed in Table 3.

Corry and O’Malley proposed that the open-cubane S_2_ conformation with O4 = OH^–^ might correspond to a higher spin configuration on the basis of DFT calculations performed in the absence of the PSII protein environment (23). The corresponding *g* value calculated directly from the modeled *J* coupling is not reported in ref. (23). We calculated the *g* value on the basis of the *J* values. The calculated *J* values do not vary significantly with basis set and functional (Table 3). Furthermore, the EPR spectra obtained from the *J* values are similar with *g* = 4.0 (Fig. 6). Nevertheless, it should also be noted that not only *S* = 5/2 but also *S* = 1/2 is the energetically lowest state even in the atomic coordinates for the open-cubane S_2_ geometry with O4 = OH^–^ used in ref. (23) (i.e. in the absence of the PSII protein environment) (Table S5), which is distinct from *S* = 5/2 being the only lowest state in the closed-cubane S_2_ conformation (Table S2). These results suggest that the open-cubane S_2_ conformation with O4 = OH^–^ corresponds to a higher spin configuration in the absence of the PSII protein environment.

**Table 3.** Calculated values for exchanging coupling *J* (cm^–1^) and *g* for the open-cubane S_2_ conformation with O4 = OH^–^ in the absence of the PSII protein environment, using the atomic coordinates in ref. (23) (i.e. W2 = OH^–^).

***J* values**	***J*_12_**	***J*_13_**	***J*_14_**	***J*_23_**	***J*_24_**	***J*_34_**	**ZFS^b^**	***g***	***S* ^c^**	**Functional**	**Basis set**	**Package**
Corry et al.	−15^a^	1^a^	3^a^	20^a^	0^a^	5^a^	total	4.0	5/2	BP86	def2-SVP (C,H) def2-TZVP (others)	ORCA
this study	−17	3	2	21	1	2	total	4.0	5/2	B3LYP	LANL2DZ (Mn,Ca) 6–31G* (others)	Jaguar

a See ref. (23).

b ZFS scheme.

c Spin *S* in the ground state.

**Fig. 7.**
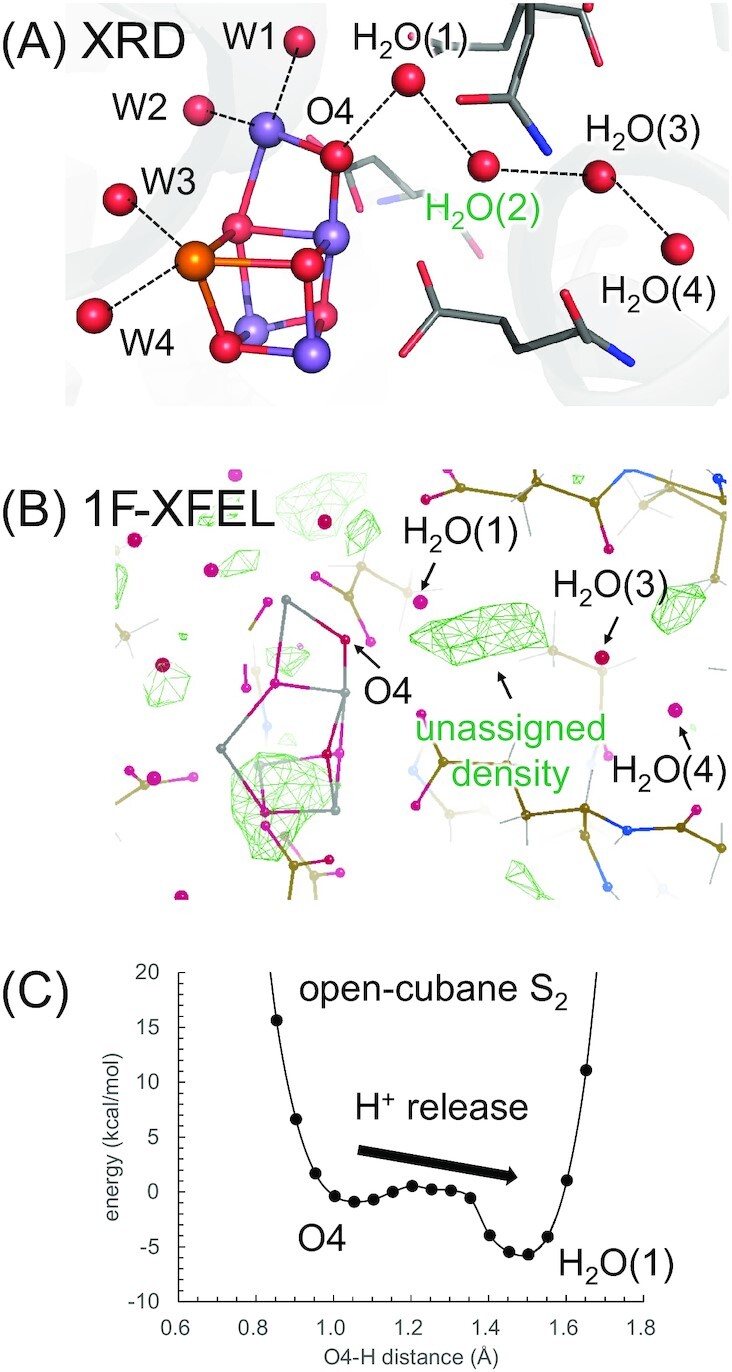
O4-water chain in the PSII protein environment. (A) Overview of the O4-water chain in the X-ray diffraction (XRD) structure (PDB code, 3ARC) (1). (B) Fo–Fc map of the 1F-XFEL structure (PDB code, 6W1P) (22). The green mesh indicates the unassigned density. (C) Energy profiles along the H-bond between OH^–^ at O4 and the adjacent water molecule H_2_O(1) for open-cubane S_2_ conformation with O4 = OH^–^ and W2 = OH^–^ [i.e. corresponding to the protonation state proposed by Corry and O’Malley (23)] in the presence of the O4-water chain, including H_2_O(2) that connects H_2_O(1) and H_2_O(3). For clarity, W539, W538, W393, and W397 (1, 36) are relabeled as H_2_O(1), H_2_O(2), H_2_O(3), and H_2_O(4), respectively.

To evaluate whether the conclusion is also relevant to the PSII protein environment, we investigated the open-cubane S_2_ conformation with O4 = OH^–^ in the PSII protein environment, using a QM/MM approach. When W2 = H_2_O, the open-cubane S_2_ conformation with O4 = OH^–^ is unstable in the presence of the H-bond network via O4 [O4-water chain: O4. . .H_2_O(1). . .H_2_O(2). . .H_2_O(3). . ., which corresponds to W539, W538, and W393. . . in refs. (1, 36), Fig. 7A], releasing the proton toward the O4-water chain (36). The present QM/MM calculation shows that even when W2 = OH^–^, the open-cubane S_2_ conformation with O4 = OH^–^ is unstable, immediately releasing the proton toward the O4-water chain (Fig. 7C) and transforming into the energetically stable low spin configuration with *S* = 1/2 (Table S5).

The second water molecule H_2_O(2) is already absent in the model complex used by Corry and O’Malley, due to the absence of the entire PSII protein environment (23). If the second water molecule H_2_O(2) were absent and the H-bond network of the O4-water chain were terminated at the first water molecule H_2_O(1), the energetically unstable OH^–^ at O4 could not release the proton toward H_2_O(1) due to significantly low p*K*_a_(H_2_O/H_3_O^+^) (= –1.7) for a single water molecule. Thus, the open-cubane S_2_ conformation with O4 = OH^–^ is an energetically unstable conformation in the PSII protein environment, having “an empty cavity” [i.e. low dielectric constant, 8.0 (23)] adjacent to “a highly deprotonatable OH^–^” at the O4 site.

The second water molecule H_2_O(2) is not explicitly assigned in recent XFEL structure in S_2_ (1F-XFEL structure) reported by Ibrahim et al. (22). However, this is not due to the absence of the density of the water molecule but due to the high disorder of the water molecule (i.e. high dielectric constant), as suggested by Suga et al. (9). Indeed, the Fo–Fc map of the 1F-XFEL structure reported by Ibrahim et al. (22) shows that an unassigned density for water molecules exists in the corresponding region (Fig. 7B). As the region with a high dielectric constant resembles bulk water rather than vacuum and their DFT calculations, where the high dielectric region was replaced with a vacant cavity (23), did not consider water dynamics appropriately, the existence of the highly deprotonatable open-cubane S_2_ conformation with O4 = OH^–^ is unlikely in the presence of the disordered water molecule in the actual protein environment. In addition, ESEEM and ENDOR data imply that the *μ*-oxo bridges of the Mn_4_CaO_5_ cluster are already deprotonated in S_2_ (and S_1_ due to the absence of the proton release during the S_1_ to S_2_ transition) (25, 55–60). They need to clarify (i) how the proton can remain bounded to O4 regardless of the H-bond formation between O4 and the proton-conducting O4-water chain (36, 37) in the S_0_ to S_1_ and even S_1_ to S_2_ transitions and (ii) how the release of the proton occurs in the S_0_ to S_1_ transition.

### Deprotonation of H_2_O at W1

**Fig. 8.**
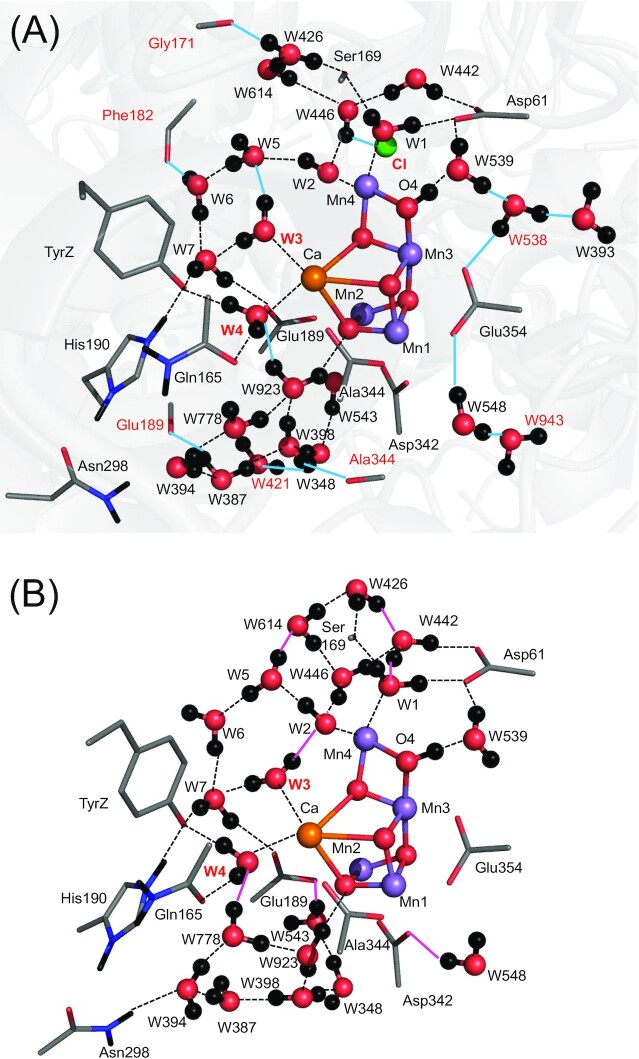
H-bond network of the open-cubane S_2_ conformation with O4 = OH^–^. (A) This study (QM/MM calculations). The protein environment is gray-colored. (B) DFT calculations by Corry and O’Malley (23) (i.e. in the absence of the PSII protein environment). Groups that show the significant difference between the two calculations are red labeled. Cyan lines indicate H-bonds that disappear in the DFT calculation mainly owing to the absence of the PSII protein environment. Pink lines indicate H-bonds that form only in the DFT calculation.

Beal et al. proposed that the closed-cubane S_2_ conformation with W1 = OH^–^ was energetically unstable in the absence of the PSII protein environment (61). However, the present study shows that W1 = OH^–^ reproduces the *g* ∼ 4 EPR spectrum in the presence of the PSII protein environment (Fig. 3). It is unclear how they appropriately modeled the protein electrostatic environment, including the H-bond network of the ligand water molecules, irrespective of the absence of the PSII protein environment, since they did not provide the atomic coordinates of the closed-cubane S_2_ conformation used in ref. (61). As far as we are aware from the atomic coordinates of the open-cubane S_2_ conformations in refs. (23, 61) (Fig. 8), it seems most likely that their conclusion was deduced from Cl^–^-depleted PSII, not from native PSII.

Cl^–^ is required in the S_2_ to S_3_ transition (62). When Cl^–^ is depleted, the S-state transition is inhibited at the S_2_TyrZ^•^ formation: that is, electron transfer occurs from TyrZ to P_D1_^•+^, but subsequent electron transfer from S_2_ to TyrZ^•^ does not occur (62). A salt bridge forms between D1-Asp61 and D2-Lys317 upon the depletion of Cl^–^ (63, 64), which may inhibit the D1-Asp61 reorientation and the proton transfer from W1 via D1-Asp61 toward the bulk region in the S_2_ to S_3_ transition (35). Upon the Cl^–^ depletion, the redox potential of S_2_/S_3_ increased significantly, making electron transfer from S_2_ to TyrZ energetically uphill (39). These may explain why the present QM/MM calculation for native PSII reproduces the *g* ∼ 4 signal in the closed-cubane S_2_ conformation with W1 = OH^–^ and the DFT calculation performed by Beal et al. for Cl^–^-depleted PSII did not.

**Fig. 9.**
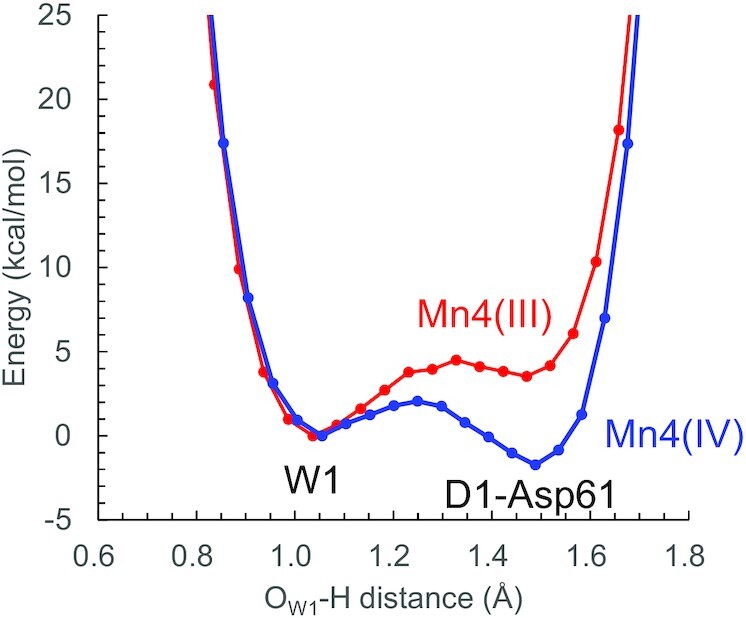
Energy profiles along the H-bond between H_2_O at W1 and D1-Asp61 in the closed-cubane S_2_ (red) and the open-cubane S_2_ (35) (blue) conformations.

A question is whether the release of the proton from H_2_O at W1 can occur in the closed-cubane S_2_ conformation in the PSII protein environment. The release of the proton from H_2_O at W1 and W2 should occur less easily in the closed-cubane S_2_ conformation with Mn4(III) than in the open-cubane S_2_ conformation with Mn4(IV). It should be noted that the release of the proton from W2 is unlikely in the closed-cubane S_2_ conformation, because the release of the proton from H_2_O at W2 toward W446 is energetically uphill in the open-cubane S_2_ conformation (28) [i.e. W2 = H_2_O (29, 30)]. In contrast, H_2_O at W1 forms a low-barrier H-bond with D1-Asp61 in the open-cubane S_2_ conformation (35) (Fig. 9), which suggests that proton transfer can occur (28, 35). The shape of the potential-energy curve for the H-bond between H_2_O at W1 and D1-Asp61 is less symmetric in the closed-cubane S_2_ conformation than in the open-cubane S_2_ conformation, which suggests that p*K*_a_(D1-Asp61) is slightly larger than p*K*_a_(W1) in the closed-cubane S_2_ conformation due to reduced Mn4(III) (Fig. 9). However, the energy barrier for proton transfer from H_2_O at W1 toward deprotonated D1-Asp61 is low, as indicated by the presence of the local energy minimum at the D1-Asp61 moiety. It seems possible that the release of the proton from H_2_O at W1 toward deprotonated D1-Asp61 occurs in the closed-cubane S_2_ conformation, which can be facilitated via proton-coupled electron transfer, as suggested in QM/MM-molecular dynamics studies (32). Because W1 is the ligand water molecule at Mn4(III) in the closed-cubane conformation, the release of the proton from W1 can facilitate oxidation of Mn4(III) to Mn4(IV) via proton-coupled electron transfer.

Fig. 3 shows that the EPR spectrum was not reproduced (due to the low energy level of the first excited spin state) when the proton of H_2_O at W1 is fully localized at the W1 moiety (W1 = H_2_O and W2 = H_2_O, Fig. 3E). It seems likely that either the migration of the proton toward the D1-Asp61 moiety (HO^–^. . .HOOC-Asp61) or the relocation of the D1-Asp61 proton toward the D1-Glu65/D1-Glu312 channel [HO^–^. . .O(OH)C-Asp61 (34, 35)] is a prerequisite for the *g* ∼ 4 signal formation.

## Conclusions

Based on all six pairwise *J* values calculated in the presence of the PSII protein environment, *g* = 4.1 is obtained exclusively for the closed-cubane S_2_ conformation with W1 = OH^–^. The result is consistent with QM/MM-molecular dynamics studies by Narzi et al. that showed the OH^–^ formation at W1 in the closed-cubane S_2_ conformation in response to the release of the proton toward D1-Asp61 (32). The shape of the simulated EPR spectrum for W2 = OH^–^ does not resemble of that for typical *g* = 4.1 signal. The simulated EPR spectrum for the closed-cubane S_2_ conformation presented in ref. (13) needs to be revisited. The *D*_4_*-z* axis in the Mn4-site-ZFS scheme is unlikely to represent the *D*-*z* axis in the total-ZFS scheme. The total-ZFS scheme seems to be more appropriate than the Mn4-site-ZFS scheme (Fig. 5).

The existence of the highly deprotonatable open-cubane S_2_ conformation with O4 = OH^–^, which was proposed on the basis of DFT calculations performed by Corry and O’Malley in the absence of the PSII protein environment (23), is energetically unlikely in the presence of the disordered water molecule in the PSII protein environment (9, 22) (Fig. 7), since their DFT models are unlikely to consider water dynamics sufficiently.

**Fig. 10.**
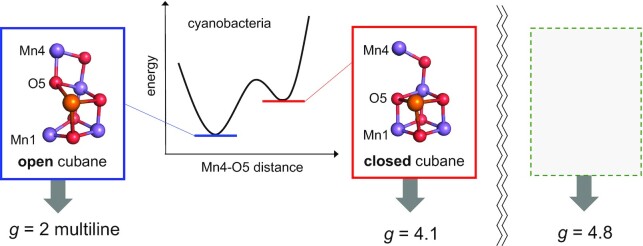
Open- and closed-cubane S_2_ conformations and corresponding EPR signals in cyanobacterial PSII. The *g* = 4.8 signal is unlikely to originate from the closed-cubane S_2_ conformation.

Although the *g* = 4.1 signal has not been reported for cyanobacterial PSII, the *g* = 4.1 signal (not the *g* = 4.8 signal) was reproduced if S_2_ is in the closed-cubane conformation even in the cyanobacterial PSII crystal structure (Fig. 3). These suggest that the *g* = 4.1 conformation, i.e. the closed-cubane S_2_ conformation, is unstable in cyanobacterial PSII, and the *g* = 4.8 conformation is unlikely to correspond to the closed-cubane S_2_ conformation (Fig. 10) [e.g. (65)].

## Funding

This research was supported by JST CREST (JPMJCR1656 to H.I.), JSPS KAKENHI (18H05155, 18H01937, 20H03217, and 20H05090 to H.I., 16H06560 and 18H01186 to K.S., and 20H05096 to H.M.), the Interdisciplinary Computational Science Program in CCS, University of Tsukuba (K.S.), and Nanotechnology Platform Program, Inst. Mol. Sci. Okazaki (JPMXP0S21MS1007 to H.M.).

## Authors' Contributions

H.I. designed research; K.S., H.M., S.N., and H.I. performed research; K.S. and H.I. analyzed data; and K.S. and H.I. wrote the paper.

## Supplementary Material

pgac221_Supplemental_File
