# Supplementary material for: Protonation structure of the closed-cubane conformation of the O2-evolving complex in photosystem II
Source: PNAS Nexus. 2022 Oct 3;1(5):pgac221. doi: 10.1093/pnasnexus/pgac221 (PMC9802176; doi:10.1093/pnasnexus/pgac221)
Supplement: pgac221_Supplemental_File [file pgac221_supplemental_file.docx]

**Supporting Information**

Protonation structure of the closed-cubane conformation of the O_2_-evolving complex in photosystem II

Keisuke Saito ^1,2^*, Hiroyuki Mino ^3^, Shunya Nishio ^1^, Hiroshi Ishikita ^1,2^*

1) Department of Applied Chemistry, The University of Tokyo, 7-3-1 Hongo, Bunkyo-ku, Tokyo 113-8654, Japan

2) Research Center for Advanced Science and Technology, The University of Tokyo, 4-6-1 Komaba, Meguro-ku, Tokyo 153-8904, Japan

3) Division of Material Science, Graduate School of Science, Nagoya University, Furo-cho, Chikusa-ku, 464-8602 Nagoya, Aichi, Japan

**Figure S1.** HOMO and LUMO of the closed-cubane S_2_ conformation. (a) W1 = OH^–^ with the *dz*^2^ axis [O4…Mn4…W2]. (b) W1 = OH^–^ with the *dz*^2^ axis [D170...Mn4...E333]. (c) W2 = OH^–^...W446 with the *dz*^2^ axis [O4…Mn4…W2]. (d) W2 = OH^–^...O5 with the *dz*^2^ axis [O4…Mn4…W2].

**Figure S2.** Estimated *g* value of 2.9 from the EPR spectrum calculated by using parameters in Pantazis et al., which is reported in Figure S5 in Supporting Information of ref 13 in the main text.

**
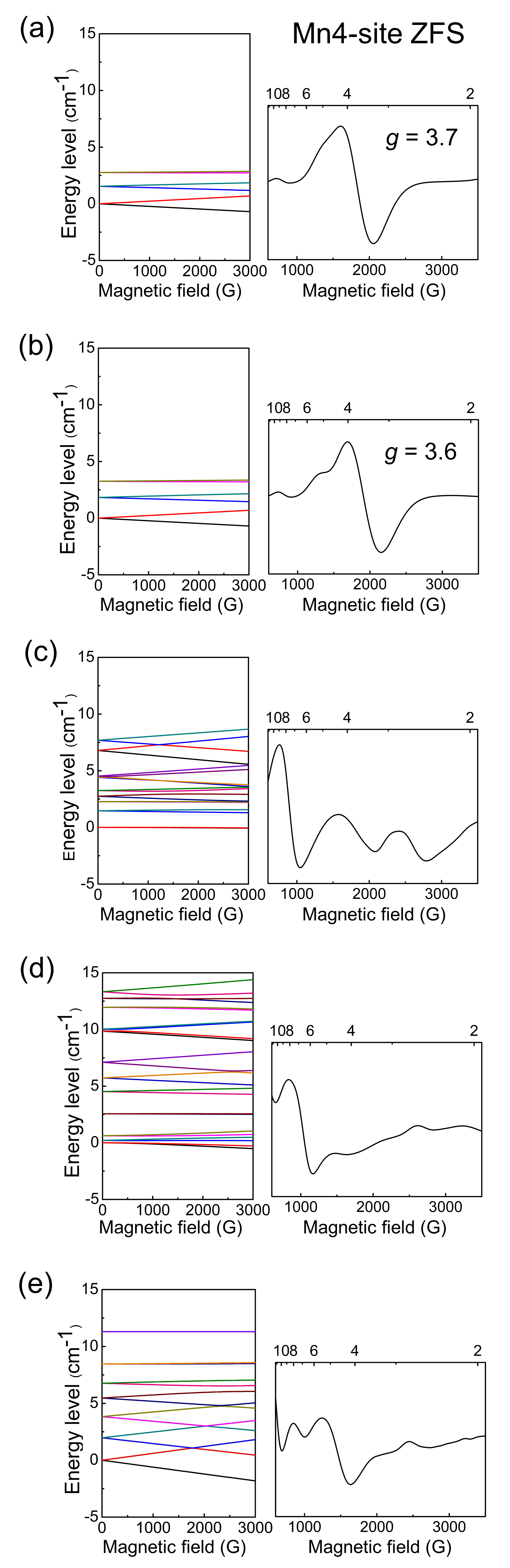
**

**Figure S3.** Simulated EPR spectra and energy level *E*_n_(***B*_0_**) obtained using the onsite ZFS tensor, ***D*_4_**, for Mn4 (i.e., the Mn4-site ZFS scheme). *E*_n_(***B*_0_**) is the energy level when ***B*_0_** is parallel to the principle *z* axis of the ***D*_4_** (*B*_0_//*D*_4,_*_z_*). (a) W1= OH^–^ and W2 = H_2_O with the Mn4-*dz*^2^ axis [D170...Mn4...E333]. (b) W1= OH^–^ and W2 = H_2_O with the Mn4-*dz*^2^ axis [O4...Mn4...W2]. (c) W1= H_2_O and W2 = OH^–^…O_W446_ with the Mn4-*dz*^2^ axis [O4...Mn4...W2]. (d) W1= H_2_O and W2 = OH^–^…O_W5_ with the Mn4-*dz*^2^ axis [O4...Mn4...W2]. (e) W1= H_2_O and W2 = H_2_O with the Mn4-*dz*^2^ axis [O5...Mn4...W1]. The EPR transitions (a-e) are calculated considering the lowest 6, 6, 18,18, and 14 sublevels, respectively. *d*_4_ = –3 cm^–1^ and *e*_4_/*d*_4_ = 0.25 were used (eq. 20).

**Table S1.** Convergence criteria of QSite used in the present study.

| **convergence criteria** | **values** |
| --- | --- |
| [SCF] |  |
| Energy change | 5.0×10^–5^ hartree |
| RMS density matrix change | 5.0×10^–6^ |
| [geometry optimization: QM region] |  |
| Maximum element of gradient | 4.5×10^–4^ hartree/bohr |
| RMS of nuclear displacement elements | 1.2×10^–3^ hartree/bohr |
| Difference between final energies from previous and current geometry optimization iterations | 5.0×10^–5^ bohr |
| RMS of gradient elements | 3.0×10^–4^ bohr |
| Maximum element of nuclear displacement | 1.8×10^–3^ hartree |
| [geometry optimization: MM region] |  |
| Energy change | 10×10^–7^ kcal/mol |
| Gradient | 0.01 kcal/(mol Å) |

**Table S2.** Adiabatic total energy *^S^E*_sc_ (cm^–1^) calculated using the optimized geometries of all possible spin configurations.

|  | **W1** | **OH^–^** | **OH^–^** | **H_2_O** | **H_2_O** | **H_2_O** |
| --- | --- | --- | --- | --- | --- | --- |
|  | **W2** | **H_2_O^b^** | **H_2_O^c^** | **OH^–^...O_W446_** | **OH^–^...O_W5_** | **H_2_O** |
| **Total spin *S*** | **Spin configuration^a^** |  |  |  |  |  |
| 13/2 | (↑↑↑↑) | 490 | 276 | 27 | 40 | 0 |
| 7/2 | (↑↑↓↑) | 253 | 229 | 129 | 191 | 261 |
| 7/2 | (↑↓↑↑) | 958 | 771 | 596 | 492 | 527 |
| 7/2 | (↓↑↑↑) | 799 | 579 | 504 | 213 | 310 |
| 5/2 | (↑↑↑↓) | 0 | 0 | 0 | 0 | 66 |
| 1/2 | (↓↓↑↑) | 809 | 603 | 481 | 389 | 364 |
| 1/2 | (↓↑↓↑) | 401 | 332 | 400 | 213 | 583 |
| 1/2 | (↑↓↓↑) | 261 | 278 | 212 | 198 | 234 |

^a^ (Mn1, Mn2, Mn3. Mn4). ^b^ *dz*^2^ axis: [D170...Mn4...E333]. ^c^ *dz*^2^ axis: [O4...Mn4…W2].

**Table S3.** Vertical total energy *^S^E*_sc_ (cm^–1^) calculated using the optimized closed-cubane S_2_ geometry of the grand-state spin configuration.

|  | **W1** | **OH^–^** | **OH^–^** | **H_2_O** | **H_2_O** | **H_2_O** |
| --- | --- | --- | --- | --- | --- | --- |
|  | **W2** | **H_2_O^b^** | **H_2_O^c^** | **OH^–^...O_W446_** | **OH^–^...O_W5_** | **H_2_O** |
| **Total spin *S*** | **Spin configuration^a^** |  |  |  |  |  |
| 13/2 | (↑↑↑↑) | 584 | 387 | 73 | 0 | 0 |
| 7/2 | (↑↑↓↑) | 262 | 228 | 166 | 255 | 320 |
| 7/2 | (↑↓↑↑) | 1056 | 887 | 480 | 465 | 549 |
| 7/2 | (↓↑↑↑) | 900 | 696 | 460 | 371 | 327 |
| 5/2 | (↑↑↑↓) | 0 | 0 | 0 | 80 | 118 |
| 1/2 | (↓↓↑↑) | 910 | 754 | 437 | 392 | 383 |
| 1/2 | (↓↑↓↑) | 435 | 385 | 391 | 517 | 635 |
| 1/2 | (↑↓↓↑) | 278 | 268 | 198 | 254 | 293 |

^a^ (Mn1, Mn2, Mn3. Mn4). ^b^ *dz*^2^ axis: [D170...Mn4...E333]. ^c^ *dz*^2^ axis: [O4...Mn4…W2].

**Table S4.** Exchanging coupling *J* (cm^–1^) calculated with the vertical approximation (i.e., using the optimized geometry of the grand-state spin configuration).

| **Ligand**  **/Mn4-*dz^2^* axis** | ***J*_12_** | ***J*_13_** | ***J*_14_** | ***J*_23_** | ***J*_24_** | ***J*_34_** | ***J*_14+24+34_** | ***S*** |
| --- | --- | --- | --- | --- | --- | --- | --- | --- |
| W1: OH^–1^, W2: H_2_O  /D170-Mn-E333 | 25.3 | 7.6 | 1.3 | 25.0 | 1.3 | -51.5 | -48.9 | 5/2 |
| W1: OH^–^ , W2: H_2_O  /O4-Mn-W2 | 24.1 | 7.9 | 1.4 | 25.1 | 4.4 | -38.4 | -32.6 | 5/2 |
| W1: H_2_O,  W2: OH^–^ (to O_W446_)  /O4-Mn-W2 | 23.7 | 8.8 | 7.7 | 20.7 | 0.5 | -14.4 | -6.2 | 5/2 |
| W1: H_2_O  W2: OH^–^ (to O_W5_)  /O4-Mn-W2 | 24.5 | 5.9 | 8.0 | 25.6 | 1.0 | -2.5 | 6.5 | 13/2 |
| W1: H_2_O, W2: H_2_O  /O5-Mn-W1 | 27.3 | 0.6 | 6.3 | 31.9 | 1.3 | 2.2 | 9.8 | 13/2 |

**Table S5.** Adiabatic total energy *^S^E*_sc_ (cm^–1^) calculated using the optimized geometries with protonated O4 (O4= OH^–^) and deprotonated O4 (O4 = O^2–^) of all possible spin configurations.

|  | **W1** | **H_2_O** | **H_2_O** | **OH^–^** | **H_2_O** | **H_2_O** |
| --- | --- | --- | --- | --- | --- | --- |
|  | **W2** | **OH^–^...O_W5_** | **OH^–^...O_W5_** | **H_2_O** | **OH^–^...O_W5_** | **H_2_O** |
|  | **O4** | **OH^–^** | **OH^–^** | **O^2–^** | **O^2–^** | **O^2–^** |
| ***J* values** |  | **Corry et al. ^b^** | **this study ^c^** |  |  |  |
| **Total spin *S*** | **Spin configuration^a^** |  |  |  |  |  |
| 13/2 | (↑↑↑↑) | 132 ^b^ | 141 ^c^ | 425 | 686 | 636 |
| 7/2 | (↑↑↓↑) | 369 ^b^ | 389 ^c^ | 523 | 460 | 437 |
| 7/2 | (↑↓↑↑) | 132 ^b^ | 131 ^c^ | 197 | 335 | 369 |
| 7/2 | (↑↑↑↓) | 213 ^b^ | 187 ^c^ | 295 | 415 | 334 |
| **5/2** | **(↓↑↑↑)** | **0** ^b^ | **2** ^c^ | 147 | 207 | 253 |
| 1/2 | (↑↑↓↓) | 360 ^b^ | 395 ^c^ | 705 | 795 | 741 |
| 1/2 | (↑↓↑↓) | 213 ^b^ | 164 ^c^ | 21 | 137 | 142 |
| **1/2** | **(↑↓↓↑)** | 9 ^b^ | 0 ^c^ | **0** | **0** | **0** |

^a^ (Mn1, Mn2, Mn3. Mn4). ^b^ Calculated from *J* values shown in Table 3 (Corry et al.: BP86/ORCA) [T. A. Corry, P. J. O’Malley. *J. Am. Chem. Soc.* **142**, 10240-10243 (2020)]) using eqs. 1-8. ^c^ Calculated with vertical approximation (this study: B3LYP/Jaguar, Table 3) using the atomic coordinates of [T. A. Corry, P. J. O’Malley. *J. Am. Chem. Soc.* **142**, 10240-10243 (2020)].
